# A novel deletion upstream of *POU3F4* in a Chinese family with X-linked deafness 2 and a literature review

**DOI:** 10.3389/fgene.2025.1641999

**Published:** 2025-10-16

**Authors:** Yanting Yang, Shengwen Huang, Yao Liu, Dan Mu, Ting Bai, Xiaosha Jing, Lingling Xing, Hongqian Liu

**Affiliations:** ^1^ Department of Medical Genetics/Prenatal Diagnostic Center, West China Second University Hospital, Sichuan University, Chengdu, China; ^2^ Key Laboratory of Birth Defects and Related Diseases of Women and Children (Sichuan University), Ministry of Education, Chengdu, China; ^3^ Department of Obstetrics and Gynecology, West China Second University Hospital, Sichuan University, Chengdu, China; ^4^ Department of Medical Genetics/Prenatal Diagnosis Center, Guizhou Provincial People’s Hospital, Guiyang, China

**Keywords:** deletions upstream of *POU3F4*, X-linked deafness 2, *POU3F4* gene, whole-genome sequencing, hearing loss

## Abstract

**Background:**

X-linked deafness 2 (DFNX2) is a rare hereditary hearing loss characterized by progressive conductive and sensorineural hearing loss and a pathognomonic temporal bone deformity. DFNX2 is caused by mutations in the coding sequence or deletions upstream of *POU3F4*. Only 12 upstream deletions of *POU3F4* associated with DFNX2 have been reported, and the precise mechanisms underlying its pathogenesis remain fully elucidated.

**Methods:**

Whole-genome Sequencing (WGS) and linkage analysis were performed to identify potential genetic etiologies. Gap-PCR and Sanger sequencing were used to validate candidate pathogenic variants and elucidate the breakpoints. Quantitative Polymerase Chain Reaction (qPCR) was conducted to evaluate the altered expression of both POU3F4 and its downstream target genes.

**Results:**

Here, we identified a novel deletion approximately 795.5 kb in length, located about 140 kb upstream of POU3F4 in a large Chinese family. All patients are hemizygous for this deletion, and the breakpoints have been confirmed to be located at GRCh37(chrX): g.81840743_82636209. Additionally, qPCR analysis demonstrated a significant reduction in the expression levels of both POU3F4 and its downstream target genes in the affected patients, which had not been reported in previous studies. We expand the spectrum of pathogenic deletions upstream of POU3F4 associated with DFNX2.

**Conclusion:**

This study provides new molecular evidence that deletions upstream of *POU3F4* can disrupt the expression of POU3F4 and its downstream target genes in humans. Our results also enhance the understanding of the pathogenic mechanisms underlying DFNX2 associated with these deletions, as well as the downstream gene networks of POU3F4.

## 1 Introduction

DFNX2 (OMIM: #304400) accounts for approximately 50% of all families with X-linked nonsyndromic deafness, which only makes up 2%–3% of hereditary hearing loss (Petersen et al., 2008; Corvino et al., 2018). In 1971, DFNX2 was first described as an X-linked condition characterized in males by profound mixed deafness, vestibular abnormalities, and congenital fixation of the stapes (Nance et al., 1971). Subsequently, in 2002, the cochlear abnormality associated with DFNX2 was classified as incomplete partition type III (IP-III) (Sennaroglu and Saatci, 2002).

The POU domain, class III, transcriptional factor 4, *POU3F4* (NM_000307.5), is located on chromosome Xq21 and encodes a transcription factor belonging to the POU-domain family, which includes a POU-specific domain and a POU-homeodomain (Mathis et al., 1992). In 1995, POU3F4, also known as BRN4, was identified as the gene responsible for DFNX2 (de Kok et al., 1995). The upstream deletions have been identified as a causative factor for DFNX2. The first report of a deletion upstream of POU3F4 in patients with X-linked mixed deafness dates back to 1971 (Nance et al., 1971). In 1996, deletions occurring 900 kb upstream of *POU3F4* were identified as a hotspot for DFNX2 (de Kok et al., 1996). However, only 12 upstream deletions of *POU3F4* associated with DFNX2 have been reported (de Kok et al., 1996; Arellano et al., 2000; Choi et al., 2013; Jiang et al., 2021; Chen et al., 2022). And the details of the underlying pathogenesis associated with these deletions remain unclear (Chen et al., 2022). Although the role of POU3F4 in the inner ear is indispensable, no transcriptional targets of POU3F4 have been thoroughly investigated. It still leaves an unresolved question regarding the functional network downstream of this transcription factor (Bernardinelli et al., 2023).

In this study, we identified a novel deletion situated upstream of *POU3F4* in a large Han family in China. Furthermore, we investigated the reduced expression of POU3F4 and the downregulation of its downstream target genes in patients with this upstream deletion, a finding that has not been reported in prior studies.

## 2 Materials and methods

### 2.1 Study participants

The Chinese family involved in this study was recruited from the Medical Genetics/Prenatal Diagnosis Centre of West China Second University Hospital, Sichuan University, Chengdu, China. The family consists of 26 members, with 5 affected individuals (all male). These patients exhibited profound congenital hearing loss and underwent comprehensive otological examinations and systematic assessments, including pure-tone audiometry and radiological examinations, in the local Department of Otolaryngology, Head and Neck Surgery (ENT Department). They sought genetic analysis to identify the underlying causes of their hearing impairment as the new generation reached reproductive age. Family history was collected by physicians from the Genetic Consulting Center. This study received ethical approval from the Institutional Review Board of West China Second University Hospital, Sichuan University. Informed consent was obtained from each participant or, for minors, their legal guardians. And the study was conducted in accordance with the Helsinki Declaration.

### 2.2 Genetic studies

We conducted Whole-genome Sequencing (WGS) using DNA obtained from patient samples as follows. Genomic DNA was extracted from peripheral blood samples utilizing the FitAmp Plasma/Serum DNA Isolation Kit (P-1004-1, Epigentek, Farmingdale, New York, United States). Sequencing was carried out on the Illumina HiSeq X system (Illumina, San Diego, California, United States). Additionally, we performed genetic linkage analysis as described in a previous study (Smith et al., 2011).

The PCR reaction was conducted using Golden Star T6 Super PCR Mix (TSE101, TSINGKE, Beijing, China) in a thermal cycler, beginning with an initial denaturation step at 98 °C for 1 min, followed by 34 cycles of denaturation at 98 °C for 1 min, annealing at 60 °C for 15 s, and extension at 72 °C for 1 min. After thermal cycling, a final extension was performed at 72 °C for 1 min, after which the samples were immediately placed on ice. PCR amplification was carried out using the ProFlex PCR System (Thermo Fisher Scientific, Waltham, Massachusetts, United States). Sequencing of the PCR products was performed on an ABI377A DNA sequencer (Applied Biosystems, Foster City, California, United States). We designed deletion-specific primers and wild-type (WT) specific primers for the amplification of the identified 795.5 kb deletion. Primers POU3F4-DEL-F and POU3F4-DEL-R were located upstream and downstream of the deletion breakpoint, respectively, while primers POU3F4-WT-F and POU3F4-WT-R were designed to be located in the deletion region. A complete list of the deletion-specific primer pairs is provided in Table 1. The PCR products were then resolved by gel electrophoresis and scored as either present or absent. Sanger sequencing was employed to elucidate the breakpoints of the deletion.

**TABLE 1:** Deletions upstream of POU3F4 were identified in DFNX2 and their associated clinical features.

Sequence information					Clinical information						Ref
Genomic position	Length	Distance upstream POU3F4	Ethnicity	Testing technique	Hearing loss			Malformations	Vestibular dysfunction	Other findings	
					Onset(m)	Severity	Type				
–	220 kb DEL	NA	NA	Southern blot analysis	NA	NA	NA	NA	NA	–	de Kok YJ, et al. (1996)
–	200 kb DEL	NA	NA	Southern blot analysis	NA	NA	NA	NA	NA	–	
–	8 kb DEL	NA	NA	Southern blot analysis	NA	NA	NA	NA	NA	–	
–	120 kb DEL	NA	NA	Southern blot analysis	NA	NA	NA	NA	NA	–	
–	30 kb DEL	NA	NA	Southern blot analysis	NA	NA	NA	NA	NA	–	
–	1,200 kb DEL	250kb	Danish	dideoxy dye-terminator cycle sequencing	NA	profound	SNHL	IP-III	EVA	Epilepsy, hyperkinetic syndrome, and goiter	Arellano B, et al. (2000)
ChrX: g. 80851535_82597832	1.74Mb DEL	165kb	Korean	aCGH	13	profound	Mixed	IP-III	NA	–	Choi BY, et al. (2013)
ChrX: g.81140313_82011100	870 kb DEL	750kb	Chinese	Nanopore + Sanger	6	profound	SNHL	IP-III	NA	–	(Jiang Y, et al., 2021)
ChrX: g.81548899_82006629	458 kb DEL	1,502 kb	Chinese	Nanopore + Sanger	5	profound	NA	IP-III	–	Mild autism	(Chen Y, et al., 2022)
ChrX: g.81806051_82292259	486 kb DEL	1,217 kb	Chinese	Nanopore + Sanger	5	profound	NA	IP-III	–	–	
ChrX: g.81807331_81887213	80 kb DEL	876 kb	Chinese	Nanopore + Sanger	5	profound	NA	IP-III	–	Atrial septal defect	
ChrX: g.81839469_82004841	165 kb DEL	1,503 kb	Chinese	Nanopore + Sanger	4	profound	NA	IP-III	–	–	
ChrX: g. 81840743_82636209	795.5 kb DEL	140 kb	Chinese	NGS + Sanger	3	profound	Mixed	IP-III	–		Our study

SNHL, Sensorineural Hearing Loss; EVA, vestibular aqueduct; IP-III, incomplete partition type III.

### 2.3 RNA extraction and quantitative real-time PCR (qPCR)

The RNA from the blood sample was extracted using Trizol (15596018, Thermo Fisher Scientific). Specifically, 1 mL of Trizol was used to solubilize the blood sample for 5 min at room temperature, followed by the addition of 0.2 mL of chloroform to facilitate phase separation for 2–3 min at room temperature. After centrifugation at 10,000 g for 10 min, the upper clear phase was mixed with 0.5 mL of isopropanol to precipitate the RNA for 10 min. The precipitated RNA was then collected by centrifugation at 10,000 g for 10 min at 4 °C. The RNA was re-extracted with phenol after resuspension and subsequently re-precipitated with ethanol. The concentration and purity of the RNA were assessed using a NanoDrop 2000 (Thermo Fisher Scientific).

We performed reverse-transcription to obtain cDNA, and qPCR was accomplished using KAPA SYBR^®^ FAST (suitable for qPCR, 2 ×, Universal) (KK4602, Roche, Basel, Switzerland) in an Applied Biosystems™ 7,500 Real-Time PCR System (Applied Biosystems). The PCR conditions were 40 cycles of denaturation at 95 °C for 5 s and annealing at 60 °C for 30 s. We analyzed results using the 2^−ΔΔCT^ method, with the expression of *GAPDH* serving as a reference gene (Livak and Schmittgen, 2001). Each reaction was repeated three times. The primers for quantitative real-time PCR (qPCR) are displayed in the list of primer pairs (Supplementary Table S1).

### 2.4 Statistical analysis

All data are expressed as arithmetic means ± SEM and analyzed with GraphPad Prism software (version 8.3.0, GraphPad Software, San Diego, California, United States). Significant differences between data sets were verified by a nonparametric test, as appropriate. A p-value <0.05 was considered statistically significant; (n) corresponds to the number of independent measurements.

## 3 Results

### 3.1 Characteristics of the clinical phenotype

**FIGURE 1:**
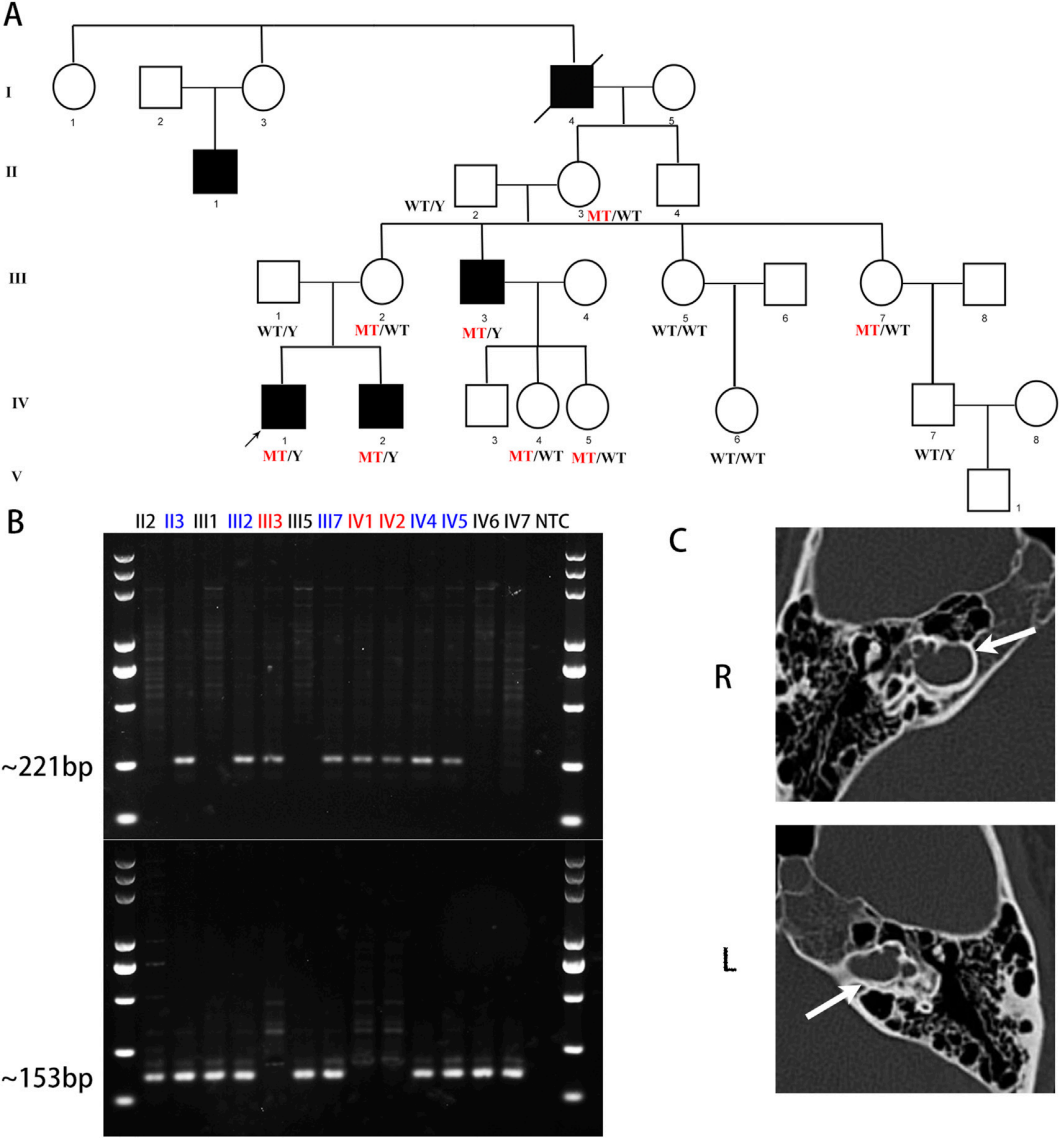
A novel deletion upstream of POU3F4 was identified in the DFNX2 family. **(A)** The pedigree of this DFNX2 family is presented. The black squares represent affected individuals with DFNX2 and a hemizygous deletion, upstream of POU3F4. **(B)** The amplicons generated by deletion-specific primers measured 221 bp, whereas the amplicons from wild-type (WT)-specific primers measured 153 bp. Individuals with a hemizygous deletion are indicated in red, while carriers with a heterozygous deletion are in blue. **(C)** Temporal bone CT scans of the patient (IV-1) revealed dilation of the internal auditory canal, with communication observed between the lateral canal and the basal cochlear turn. The white arrows indicate the internal auditory canal.

IV-1 and IV-2 were diagnosed with profound bilateral mixed hearing loss at the ENT department. IV-1, now 26 years old, received his hearing aids at the age of 3. Despite being able to engage in social conversations without experiencing tinnitus or vestibular symptoms, his speech and communication skills remain impaired. IV-2, currently 16 years old, received hearing aids at the age of 6. With the assistance of speech and language therapy, IV-2 has shown significant improvement in his speech and communication abilities. He now attends a mainstream high school, excels academically, and denies experiencing tinnitus or vestibular symptoms. At the age of 15, his speech test results in both quiet and noisy environments were deemed satisfactory. Both IV-1 and IV-2 underwent presurgical evaluations, including pure-tone audiometry and radiological examinations. However, due to the considerable time elapsed since their treatment, complete clinical data are no longer accessible. Only the temporal bone CT scans of IV-1 were obtained. The radiological images demonstrate symmetrical cochlear hypoplasia and abnormal communication between the bottom of the internal auditory canal and the vestibule, which are characteristic of DFNX2 (Figure 1C). These characteristics are all consistent with the previously reported probands harboring deletions upstream of POU3F4 (Table 1). In previous reports, one subject was described by Arellano et al. as having bilateral vestibular areflexia (Arellano et al., 2000), and one subject was described by Chen et al. as having mild autism (Chen et al., 2022) (Table 1). However, neither IV-1 nor IV-2 reported any vestibular dysfunction or manifestations related to the autism spectrum.

After gathering information regarding the family history, we constructed a pedigree chart (Figure 1A). The chart illustrates additional affected males (II-1 and III-3) who also experience profound bilateral hearing loss. Adult females in the family denied any hearing impairments. Notably, individuals II-3 (80 years old) and III-2 (50 years old) assert having normal hearing but have declined additional testing. The family exhibits a classic X-linked inheritance pattern.

### 3.2 Identification of a novel deletion in the upstream of *POU3F4*

**FIGURE 2:**
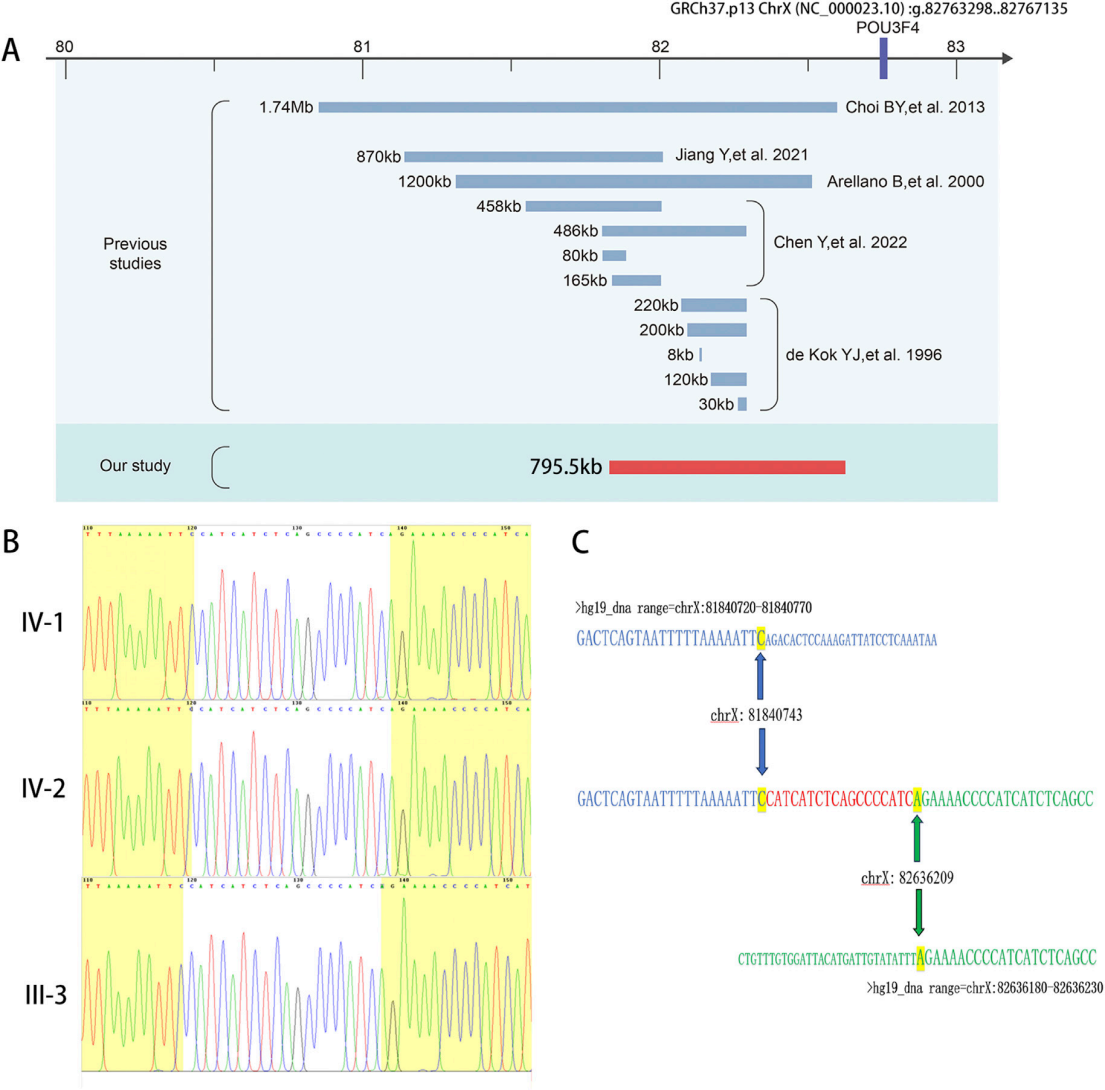
Genetic analysis of this novel 795-kb deletion located upstream of the POU3F4 gene. **(A)** A schematic illustration of the upstream region of POU3F4 is presented, displaying the deletions identified in our study as well as those reported previously. **(B) **Sanger sequencing revealed 18-bp novel added nucleotides (CAT​CAT​CTC​AGC​CCC​ATC) in the breakpoint region. The nucleotides that matched the reference sequence are highlighted in yellow. **(C)** Alignment of the sequenced junctions with the reference genome sequence. Proximal and distal reference sequences are shown in green and blue, respectively. The junction is shown in red. Distal (top, blue) and proximal (bottom, black) sequences were aligned against the junction sequence (middle).

To ascertain a genetic diagnosis, we performed Trio whole-exome sequencing (Trio-WES) on the family members of the siblings (IV-1, IV-2, III-1, and III-2). The results were negative. Considering the evident X-linked inheritance pattern in the pedigree, we subsequently conducted Trio whole-genome sequencing (Trio-WGS) and genetic linkage analysis to explore potential candidate molecular diagnosis. Remarkably, we identified a 795.5 kb deletion located at GRCh37 (chrX): g. 81840740-82636210, approximately 140 kb upstream of the *POU3F4* gene (Figure 2A), which is known to be the causative gene for DFNX2. The large deletion was absent from gnomAD, Database of Genomic Variants (http://dgv.tcag.ca/dgv/app/home), or DECIPHER (https://decipher.sanger.ac.uk/browser).

Currently, only 12 deletions upstream of *POU3F4* have been described in previous reports (de Kok et al., 1996; Arellano et al., 2000; Choi et al., 2013; Jiang et al., 2021; Chen et al., 2022). We conducted a comprehensive review of the previously reported deletions upstream of POU3F4 and the identified deletions upstream of *POU3F4* (Table 1). The deletions associated with DFNX2 exhibited variable sizes, ranging from 8 kb to 1.74 Mb, with approximate locations 140 kb–1,503 kb upstream of *POU3F4* (Figure 2A). Notably, there is no common overlapping region among these deletions. Remarkably, our investigation revealed that the downstream breakpoint of the novel deletion we detected is the closest to the *POU3F4* gene reported thus far. Our findings contribute to broadening the spectrum of pathogenic deletions associated with DFNX2, located upstream of *POU3F4*.

To assess the presence of a genomic deletion in the family, we performed polymerase chain reaction (PCR) analysis. Electrophoresis confirmed successful amplification of the junction sequence (Figure 1B). The male patients (III-3, IV-1, and IV-2) only showed the amplicons of deletion-specific primers (221bp) (Figure 1B). And tested female family members, II-3, III-2, III-7, IV-4, and IV-5, showed both the amplicons of deletion-specific primers (221bp) and amplicons of WT-specific primers (153bp) (Figure 1B). We consider these five persons as X-linked female carriers with this heterozygous deletion. We also researched the junction sequence with amplicons of deletion-specific primers by Sanger sequencing (Figure 2B). The results of the junction sequence indicated that the deletion is actually located at GRCh37. p13 ChrX (NC_000023.10): g. 81840743_82636209. Notably, the sequencing data revealed the presence of 18 novel nucleotides (CAT​CAT​CTC​AGC​CCC​ATC) added at the junction points (Figure 2C). This finding supports the hypothesis that this deletion may be the genetic cause of the observed phenotype in this family.

### 3.3 The 795.5 kb deletion upstream of the *POU3F4* impairs its expression

**FIGURE 3:**
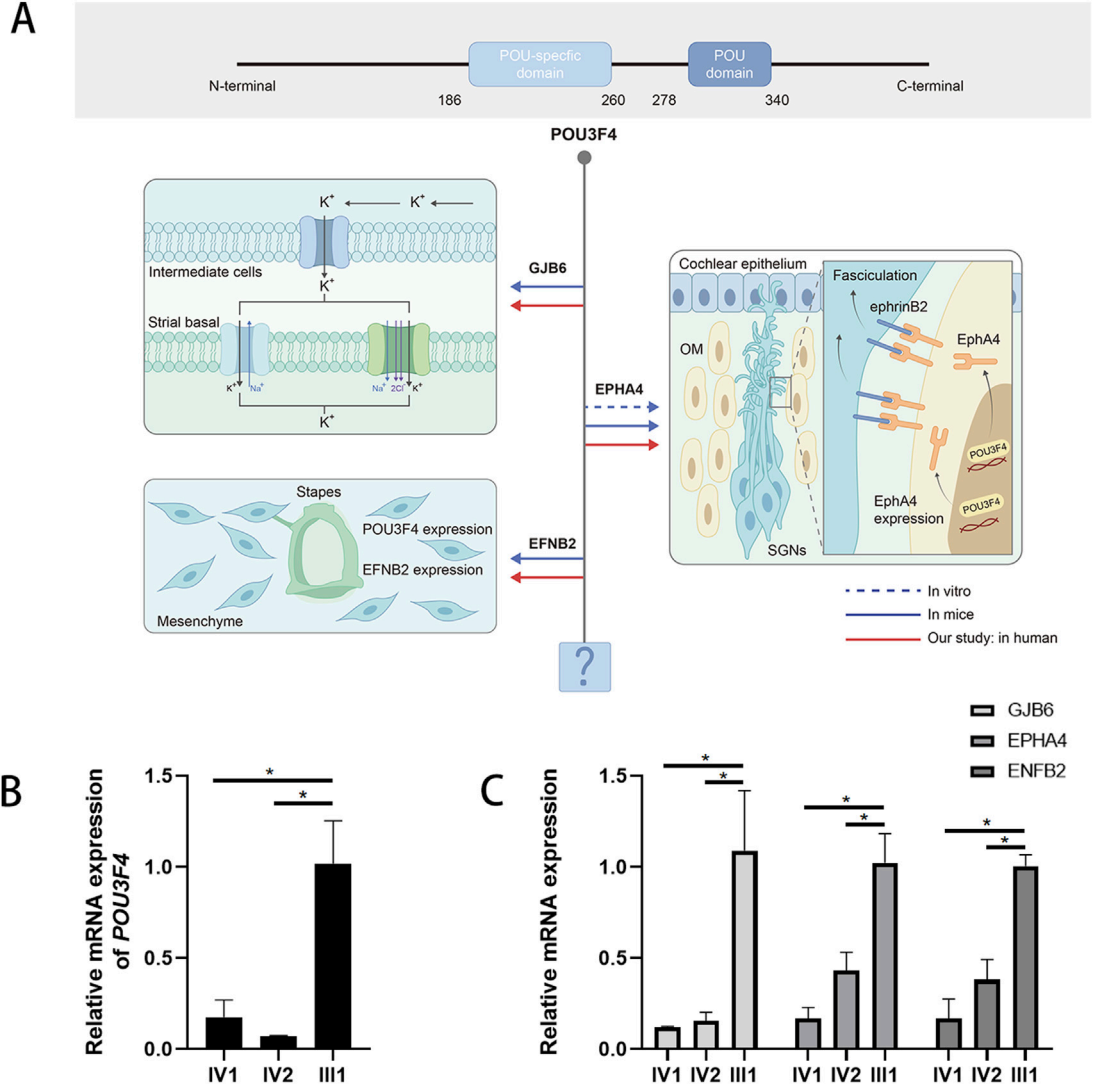
The deletion upstream of the POU3F4 we detected impairs the expression of POU3F4 and its downstream genes. **(A)** Partial mapping of the transcriptional target network of POU3F4: integrating findings from previous studies with our current research. **(B)** The result of qPCR shows a sharp decrease in the mRNA level of the affected brothers compared to their healthy father. Three independent experiments were performed. *indicate the p-value <0.05 (nonparametric test; error bars, mean ± SEM). **(C)** The result of qPCR also shows that the relative expression levels of GJB6, EPHA4, and EFNB2 mRNA in the affected patients were significantly lower compared to normal people. Three independent experiments were performed. * indicate the p-value <0.05 (nonparametric test; error bars, mean ± SEM).

To determine whether the deletion (NC_000023.10: g. 81840743_82636209del) located upstream of *POU3F4* affects the mRNA expression of the *POU3F4* gene, we conducted a quantitative real-time PCR (qPCR) assay using RNA extracted from the peripheral leukocytes of the patients. The relative expression levels of *POU3F4* mRNA in affected siblings (IV-1 and IV-2) with the upstream deletion of POU3F4 that we detected were significantly lower compared to the male normal control (III-1) (Figure 3B).

### 3.4 Upstream deletion also affected the expression of downstream targets of POU3F4

To gain a deeper understanding of the deletion identified upstream of *POU3F4*, we also assessed the mRNA expression levels of *GJB6*, *EPHA4*, and *EFNB2*(*Ephrin-B2*), which are known to be influenced by POU3F4 during inner ear development (Figure 3A). The relative expression levels of *GJB6*, *EPHA4*, and *EFNB*2 mRNA in the affected patients were also significantly lower compared to those of normal people (Figure 3C). According to previous studies, the impact of POU3F4 on downstream genes has been demonstrated *in vitro* and in mice (Figure 3A). Our findings further substantiate the effect of the deletion detected upstream of *POU3F4* on the expression of its downstream genes. Additionally, the influence of POU3F4 on its downstream genes has been observed in humans with DNFX2 for the first time. Meanwhile, these findings also provide new evidence to investigate the functional network downstream of POU3F4.

Thus, the novel deletion (NC_000023.10: g. 81840743_82636209del) at approximately 140 kb upstream of the *POU3F4* gene disrupts POU3F4 expression, leading to impaired function. This negative function further impacted other important proteins in inner development, potentially culminating in the DFNX2 phenotype. Collectively, these findings strongly indicate that the identified deletion upstream of *POU3F4* could serve as the molecular basis for the hearing loss observed in this family.

## 4 Discussion

In a large Han family with DFXN2, we detected a novel 795.5 kb deletion on ChrX (NC_000023.10: g. 81840743_82636209), situated approximately 140 kb upstream of *POU3F4*. Following a negative result from whole-exome sequencing (WES), we conducted whole-genome sequencing (WGS) and genetic linkage mapping using SNP genotypes extracted from the WGS data to investigate candidate molecular diagnosis. In our study, we first demonstrated that deletion upstream of *POU3F4* impacted the mRNA expression of *POU3F4*. Furthermore, the decreased RNA expression levels of downstream targets of POU3F4 were also first observed in patients with this deletion that we identified. Based on our experimental data, we propose that the 795.5 kb deletion upstream of *POU3F4* may lead to DFNX2 by impairing the expression of both POU3F4 and its downstream functional network.

In our study, we also detected the presence of 18 novel nucleotides (CAT​CAT​CTC​AGC​CCC​ATC) inserted at the junction points. This finding is consistent with the previous report. In the study of Jiang Yi et al., non-homologous end joining (NHEJ), an important type of genome rearrangement (Ja et al., 2007), was identified as a mechanism for the deletion in the upstream region of *POU3F4* (Jiang et al., 2021). Notably, NHEJ typically results in the loss of several nucleotides at each end of the DNA break (Hhy et al., 2016). The 18-bp sequence (CAT​CAT​CTC​AGC​CCC​ATC) we detected is the “information scar” at the joining points. It suggested that NHEJ may be the causal pathway for the novel 795.5 kb deletion located at approximately 140 kb upstream of *POU3F4*.

The *POU3F4* gene, approximately 8,907 bp in length, is located on the X chromosome at the Xq21.1 locus and consists of a single coding exon that spans 1,506 bp (de Kok et al., 1995). In 1997, the first deletion upstream of POU3F4 in patients with X-linked mixed deafness was reported (Nance et al., 1971). Since then, 12 deletions upstream of POU3F4 associated with DFNX2 have been reported. These deletions exhibit different lengths and different distances from POU3F4. And there is no common overlap region among these deletions (Figure 2A). This phenomenon might mean that the underlying pathogenic mechanism is not as same as general copy number variants (CNVs). According to the previous study, the human *POU3F4* gene is located in a 3-Mb gene desert region enriched in highly conserved non-coding regions (HCNRs), which are enriched in cis-regulatory elements (Alonso et al., 2009). For the tight regulation of gene expression in time and space in development, core promoters close to transcription start sites must interact with noncoding regulatory elements in their vicinity, termed enhancers (Robson et al., 2019). Enhancers and promoters communicate across large genomic distances through direct physical contact via chromatin folding, which is responsible for chromosome conformation (Robson et al., 2019). Deletions upstream of *POU3F4* might affect chromosome conformation, thereby impact the function of cis-acting elements and contributing to the pathogenic mechanism. Further studies are needed to investigate the characterization and function of this region.

Meanwhile, the functional network downstream of POU3F4 has not been fully characterized so far (Bernardinelli et al., 2023). Loss of function of POU3F4 disrupts the assembly and localization of gap junction proteins, specifically connexin 26 (Cx26) and connexin 30 (Cx30), at the cell borders of cochlear supporting cells, thereby affecting the endolymphatic potential (EP) in the inner ear (Kidokoro et al., 2014). Additionally, Coate et al. demonstrated that the expression of EPHA4, a receptor tyrosine kinase, is decreased in Pou3f4 knockout (KO) mice, and that POU3F4 binds to the regulatory elements of EphA4 (Raft et al., 2014). EPHA4 is known to regulate spiral ganglion axon fasciculation, which is essential for appropriate auditory innervation (Raft et al., 2014). Furthermore, POU3F4 and the Eph receptor transmembrane ligand Ephrin-B2 exhibit a common spatiotemporal expression pattern during the organogenesis of the middle and inner ear, providing evidence for the role of POU3F4 in the development of the bony tissue surrounding the vestibular labyrinth (Raft et al., 2014). In our study, we directly confirmed the expression alteration of this transcriptional target by real-time PCR analysis in DNFX2 patients. Our findings not only confirm the pathogenicity of the identified deletion but also provide additional evidence for the further construction of downstream gene networks.

Previous reports on upstream deletions of POU3F4 lacked mRNA-level validation or verification. This limitation stemmed from the challenging nature of constructing animal or cell models for such deletions, which are situated in noncoding regions and range from 8 kb to 1.74 Mb in size. Despite the advancements in gene editing through CRISPR-Cas9, precise deletion of large DNA fragments, spanning from kilobases to megabases, remains a persistent challenge (Li et al., 2023). Choi et al. showcased the potential for accurate long genomic sequence deletions and replacements; however, they highlighted a relatively low deletion efficiency, with only about a 2% success rate observed for a 10 kb deletion (Choi et al., 2022). In humans, the *POU3F4* gene is predominantly expressed in the nervous system. Nevertheless, mRNA of *POU3F4* has also been detected in whole blood in the BioGPS database (BioGPS - your Gene Portal System). Quantitative real-time PCR is commonly employed in gene expression profiling (Zainal Ariffin et al., 2022). Hence, we utilized a qPCR assay with RNA extracted from peripheral leukocytes of patients to assess whether upstream deletion of *POU3F4* impacts *POU3F4* gene expression. Additionally, we performed qPCR to validate the expression of these downstream genes of POU3F4, further confirming the deleterious effect of the detected deletion. However, the specific pathogenic mechanism of the upstream deletion of the POU3F4 gene remains unclear and needs further study.

## 5 Conclusion

Our study identified a novel deletion located at approximately 140 kb upstream of *POU3F4* in a large Han family with DFXN2. Our findings expand the spectrum of pathogenic deletions associated with DFNX2 that occur upstream of the POU3F4. We first revealed that the deletion upstream of POU3F4 impacted the mRNA expression of POU3F4 and its downstream genes in patients with this deletion. Our work presented more detailed information on the pathogenesis of deletion upstream of *POU3F4* and provided further evidence to the understanding of downstream gene networks of POU3F4.

## Data Availability

For the considerations about the security of human genetic resources and the confidentiality of participant, the data is not publicly available, but can be applied from the corresponding author on reasonable request.
