# Supplementary material for: A novel deletion upstream of POU3F4 in a Chinese family with X-linked deafness 2 and a literature review
Source: Front Genet. 2025 Oct 16;16:1641999. doi: 10.3389/fgene.2025.1641999 (PMC12571451; doi:10.3389/fgene.2025.1641999)
Supplement: Supplementary file 1 [file Table1.docx]

**Supplementary Table 1.** Overview of the information of primer sequences in Sanger sequencing and qPCR

| **Primers** | **Sequences** |
| --- | --- |
| *POU3F4*-DEL | F 5' - TGAATCGTTTCCTATGTTGAG -3'  R 5' - TTTGTATCCTGAGACTTTGCT -3' |
| *POU3F4*-WT | F 5' - AGCCTGGCTTTAGGAAGGAA -3'  R 5' - TTTGGCAGATCTCCAGGGTA -3' |
| *POU3F4*-hum-qPCR | F 5' - TGCGTGTCTGGTTCTGTAATC -3'  R 5' - GTGTGCGAATAAACCTCATGC -3' |
| *GJB6*-hum-qPCR | F 5' - GATCTTGCCCTCATTCTTTGG -3'  R 5' - GATCCACACCTTCCCGATG -3' |
| *EPHA4* -hum-qPCR | F 5' - GGAGAACTTGGGTGGATAGC -3'  R 5' - TTGGTAGGTTCGGATTGGTG -3' |
| *EFNB2* -hum-qPCR | F 5' - GAATTCAGCCCTAACCTCTGG -3'  R 5' - ATCTTCATGGCTCTTGTCTGG -3 |
| *GAPDH* -hum-qPCR | F 5' - AATCCCATCACCATCTTCCAG -3'  R 5' - AAATGAGCCCCAGCCTTC -3 |
